# A Novel Ocular Tonometry Task Trainer

**DOI:** 10.7759/cureus.31366

**Published:** 2022-11-11

**Authors:** Jeffrey Heimiller, Lawrence Stack, Joseph Sikon, Ryan Walsh, Charles Lei

**Affiliations:** 1 Department of Emergency Medicine, Vanderbilt University Medical Center, Nashville, USA

**Keywords:** task trainer, procedural skills training, intraocular pressure, ophthalmology, emergency medicine, emergency medicine training, tonometry, task-trainer

## Abstract

The measurement of intraocular pressure via ocular tonometry is a skill necessary for the evaluation of emergency department patients with ocular complaints. Accurate results inform the use of time-sensitive medications or invasive procedures. We sought to develop and evaluate an affordable, realistic, and reproducible task trainer to allow Emergency Medicine residents and medical students to practice tonometry.

We placed an angiocatheter into the vitreous chamber of a swine eye through the optic nerve stump and sealed it with a purse string suture and cyanoacrylate glue. This allowed us to connect intravenous extension tubing and use a saline-filled syringe to repeatedly adjust intraocular pressure in real time. Optionally, this model can be mounted in a polystyrene foam mannequin head to enhance realism and facilitate practice.

The task trainer was implemented in medical student and Emergency Medicine resident education at Vanderbilt University Medical Center. Thirty-six learners participated in the study, all of whom completed pre-course and post-course surveys. Among all learners, the mean comfort with performing tonometry improved significantly (3.26 to 7.64 {Z = -4.95, p < 0.005}). The mean confidence in the accuracy of measurements also increased (3.11 to 7.56 {Z = -4.8, p < 0.005}). On a 10-point scale, learners felt this task trainer was highly helpful in increasing their comfort with and the ability to perform tonometry (mean 9.19 {SD 1.19}).

We have developed a low-cost and easily constructed ocular tonometry task trainer that resulted in significant improvement in learner comfort and confidence.

## Introduction

Measuring intraocular pressure (IOP) by tonometry is a vital skill for clinicians caring for patients with ocular injury or pathology. Decisions on therapeutic interventions, such as administering IOP-lowering medications or performing a lateral canthotomy and cantholysis, are influenced by IOP measurements. Diagnosis and treatment decisions, therefore, require accurate measurements [1,2]. Emergency medicine (EM) physicians perform this procedure less frequently than optometrists or ophthalmologists, and novice clinicians may be uncomfortable when performing tonometry for the first time due to fear of causing injury or pain. Commercially available task trainers are costly, and we are not aware of any commercial or do-it-yourself trainers that accurately facilitate tonometry training [3-6]. Foley catheter balloons and marbles have been used, but these do not demonstrate the relevant anatomy with accurate tactile feedback. We have developed, to our knowledge, the first non-commercial, inexpensive, and anatomically accurate tonometry task trainer. Using swine eyes, our trainer accurately simulates ocular tissue and allows for real-time IOP modification. We propose that this trainer would increase the confidence of novice and intermittent tonometry practitioners in the skill and accuracy of their IOP measurements. This model was previously presented as a platform presentation at the International Meeting on Simulation in Healthcare, January 25, 2021, virtual conference.

## Technical report

The model fundamentally consists of a swine eye with an angiocatheter inserted through the optic nerve sheath into the vitreous chamber. 

Model creation

**Figure 1:**
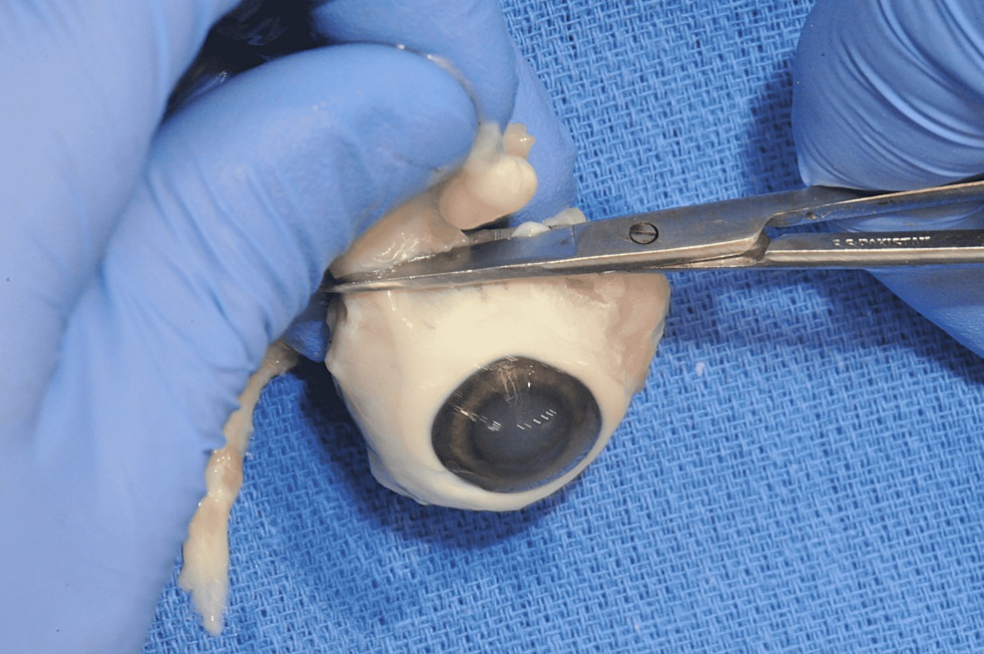
Iris scissors being used to remove excess extraocular tissue, such as muscle and connective tissue

To create the model, excess extraocular tissue adherent to the globe was first removed from the swine eyes using a pair of iris scissors (Figure 1).

**Figure 2:**
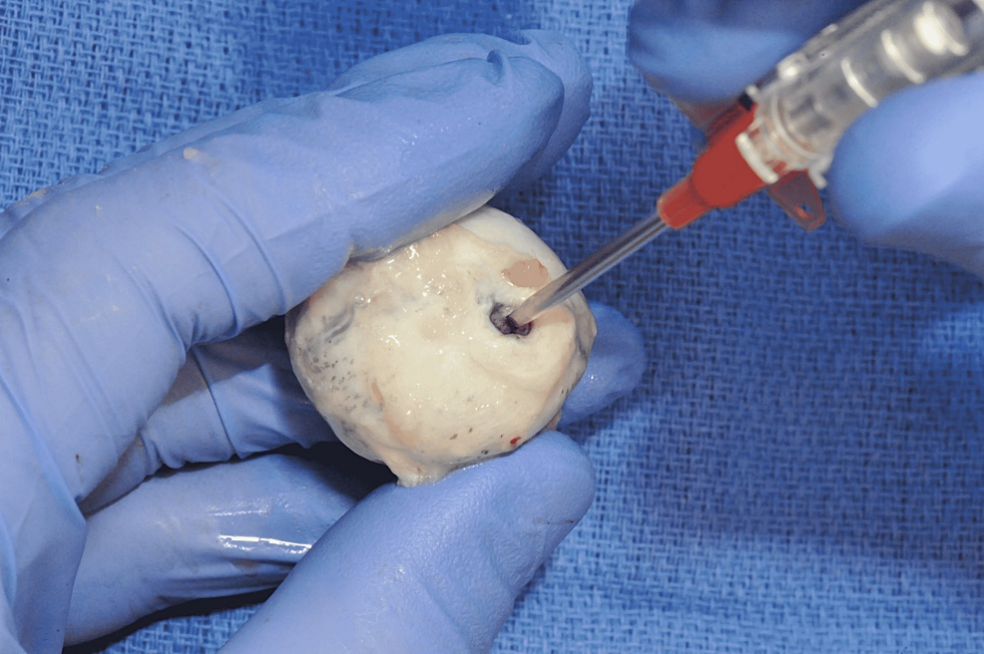
Insertion of the angiocatheter into the optic nerve stump

A 14 gauge angiocatheter was then inserted through the optic nerve stump into the vitreous (Figure 2). A loss of resistance was felt when the needle pierced the vitreous body. The angiocatheter was advanced into the vitreous and the needle was withdrawn.

**Figure 3:**
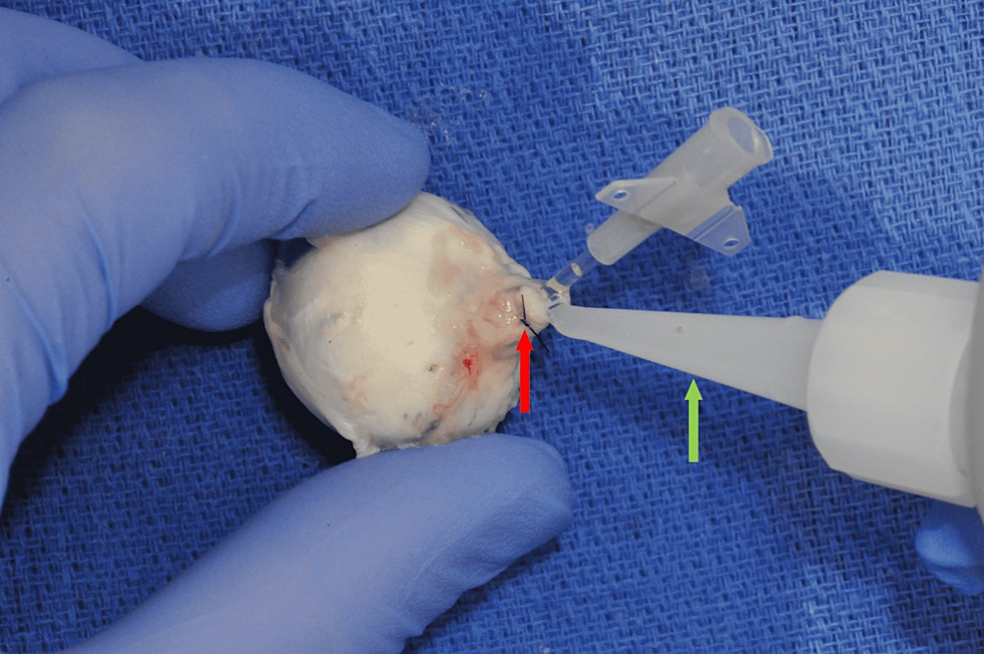
Application of cyanoacrylate glue to seal the angiocatheter to the optic nerve stump, which has been tightened with a purse string suture Red arrow: purse string suture in optic nerve stump; Green arrow: cyanoacrylate glue applicator

The angiocatheter was then secured in place using two sets of purse string sutures (5-0 nylon) tied circumferentially around the optic nerve stump. The rim of the stump was sealed to the angiocatheter using a thin bead of viscous cyanoacrylate glue (Figure 3). A cyanoacrylate glue accelerator was used to expedite drying time. 

**Figure 4:**
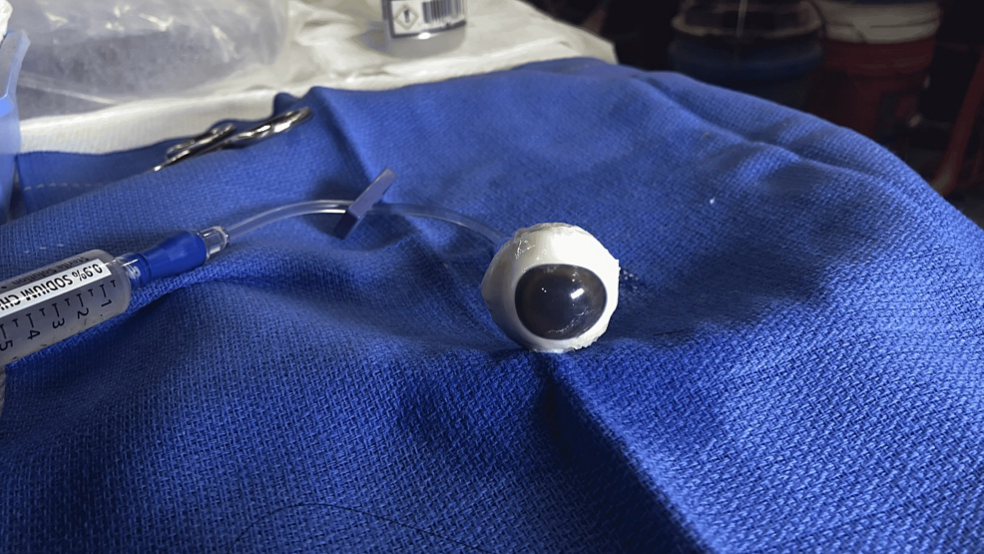
Fully assembled model with extension tubing and syringe attached

A saline-filled syringe attached to intravenous (IV) extension tubing was then connected to the angiocatheter (Figure 4). A small volume of saline (1-2 mL) was repeatedly injected and withdrawn from the vitreous (about five times) to disrupt the vitreous and allow for easy flow of saline into and out of the eye. Once this was completed, the eye was pressurized using the saline-filled syringe and IV tubing. Between learners, fluid was injected or withdrawn to vary the measured IOP.

**Figure 5:**
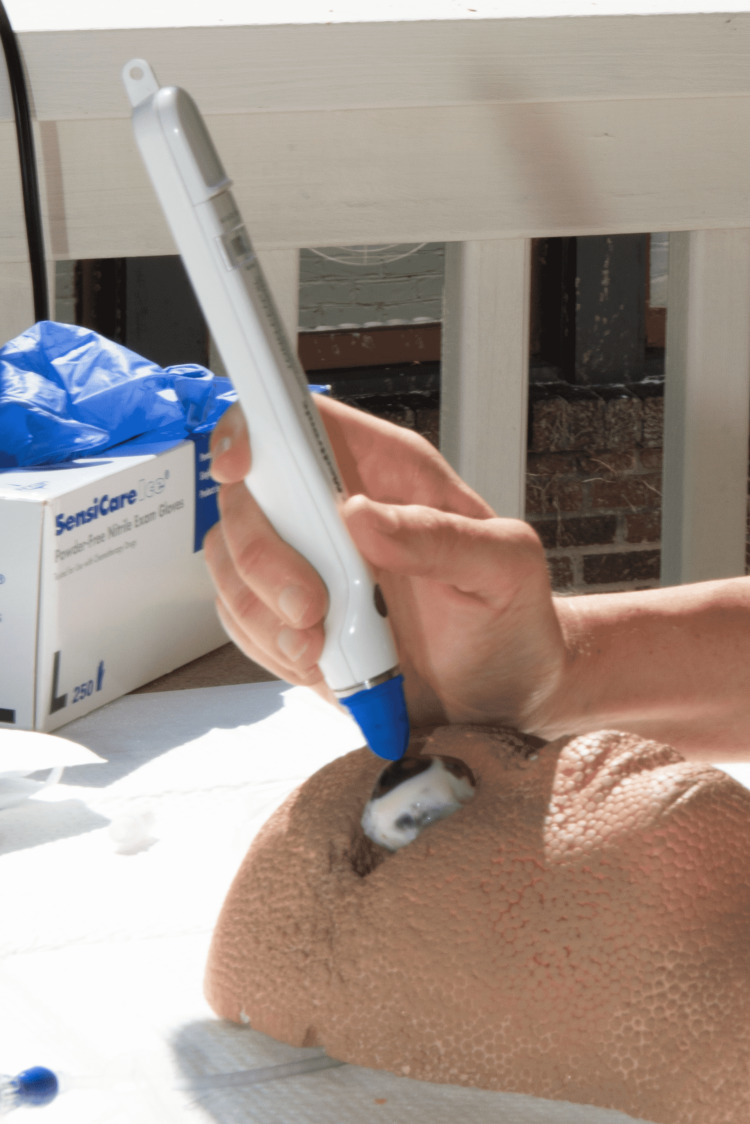
Model mounted in Styrofoam head during tonometry training

For the intern training session, the swine eye was mounted in a Styrofoam mannequin head to better recreate the clinical exam (Figure 5). A coronal cut was made through a three-dimensional mannequin head, leaving only the anterior half. The orbit was enlarged with a rotary tool. A hole was drilled posteriorly from the orbit to allow the IV tubing to exit from the back of the mannequin head. Incorporation of this mannequin head is optional, and it was not used during our medical student sessions.

Model use and assessment

This model was incorporated into our EM medical student clerkship procedure lab (third- and fourth-year students) and our EM intern introductory training. Recruitment was consecutive for the duration of the study (June 2019 - June 2020) and participation was voluntary. Participants completed a pre-course survey, providing data on their basic demographics, procedural knowledge, comfort with performing the procedure, and confidence in their ability to obtain accurate tonometry results. Learners were then given instruction on the importance of IOP measurement and the use of the tonometer, followed by practice using the swine eye model. Each participant was allowed to practice IOP measurement as many times as they wished, although we noticed that none chose to practice more than three times. Saline was injected or withdrawn between learners so that each learner would need to obtain a new measurement. Instructors were available to independently confirm results, but most learners felt comfortable relying on the tonometer's reported confidence index. Afterward, they completed a post-course survey in which they reported their post-session comfort and confidence in performing tonometry as well as their perceived utility of the model. Changes in learner comfort and confidence were analyzed using the Wilcoxon Signed-Rank test. The Vanderbilt University Medical Center Institutional Review Board reviewed this study and declared it exempt. 

Results

A total of 36 learners were enrolled in the study (13 EM interns and 23 medical students). One hundred percent (36/36) of participants completed the pre-course and post-course surveys. Pre-course surveys revealed that 44% (16/36) of learners had never received training on tonometry, 28% (10/36) had never seen it performed on a patient, and 50% (18/36) had never performed the procedure. The mean comfort with performing this procedure had a statistically significant increase among all learners from 3.26 to 7.64 (Z = -4.95, p < 0.005). Subjects’ mean confidence in the accuracy of their measurements also increased significantly from 3.11 to 7.56 (Z = -4.8, p < 0.005). When separated into specific groups (medical students versus interns), improvements in mean comfort with performing tonometry and mean confidence in the accuracy of their measurements maintained statistical significance. Lastly, on a 10-point scale, the mean participant rating on how helpful participants found this task trainer in increasing their comfort with and ability to perform tonometry was 9.19 (SD 1.19).

## Discussion

Intraocular pressure measurement by tonometry is a critical skill for EM physicians. The results of this procedure guide time-sensitive decisions to treat vision-threatening conditions such as glaucoma or orbital compartment syndrome, so results must be reliable [1,2]. Unfortunately, financial constraints of training programs and the lack of available and accurate commercial or do-it-yourself models largely limits the practice of this skill prior to performance on patients.

We therefore developed a low-cost tonometry task trainer that simulates the tactile sensation of performing IOP measurement on living tissue. We incorporated this task trainer into our regularly scheduled medical student procedural training, accompanied by a brief didactic session describing tonometry. We also included it in our annual EM intern ophthalmology boot camp, replacing the prior Foley catheter-based model among a rotation of ophthalmology skills stations [7]. Our task trainer was well-received by our cohort of learners. Training using our model led to statistically significant improvements in learner comfort with performing the procedure and learner confidence in their ability to obtain accurate IOP measurements. Although this effect could be contributed in part to the accompanying didactic session, learners specifically rated the model as highly helpful.

We encountered some technical difficulties in using these models. During long education sessions, the eyes became desiccated and tonometry measurements became inaccurate. This was mitigated somewhat by rinsing saline over the eyes between learners or intermittently soaking them in saline. Additionally, the seal afforded by the suture and glue often failed after multiple learners, limiting the amount of pressurization we could achieve. We, therefore, found it best to treat this as a consumable model and prepare several eyes for each educational session. 

In this study, our participant cohort was small and relatively inexperienced, which may have magnified our findings. In addition, we only evaluated self-reported comfort and confidence in performing tonometry as well as the perceived helpfulness of the task trainer. Future study directions include determining if our task trainer is effective at increasing the comfort and confidence of more experienced learners and assessing its ability to improve patient outcomes, such as obtaining more accurate IOP measurements on actual patients or decreasing patient discomfort during tonometry. It would also be useful to determine whether the added fidelity of nesting this model in a manikin head improves learner engagement or outcomes.

## Conclusions

By inserting an angiocatheter into a lightly-modified swine eye, we have created a low-cost model for training intraocular pressure measurement by tonometry. It was well-received by our learner cohort, which consisted of EM interns and medical students on their EM rotation. Survey data from these learners demonstrated statistically significant increases in learner comfort and confidence in the procedure following a training session featuring this task trainer. Our learners rated this task trainer as very helpful, and we hope that other training programs will also find it useful. 

## References

[1] Carrim ZI, Anderson IW, Kyle PM (2007). Traumatic orbital compartment syndrome: importance of prompt recognition and management. Eur J Emerg Med.

[2] Walker RA, Adhikari S (2022). Eye emergencies. Tintinalli's Emergency Medicine: A Comprehensive Study Guide.

[3] Austin PE, Ljung M, Dunn KA (1995). A new model for teaching corneal foreign body removal. Acad Emerg Med.

[4] Kong R, Kaya DP, Cioe-Pena E, Greenstein J (2018). A low fidelity eye model for lateral canthotomy training. Afr J Emerg Med.

[5] Lichtenberger JP, Tatum PS, Gada S, Wyn M, Ho VB, Liacouras P (2018). Using 3D printing (additive manufacturing) to produce low-cost simulation models for medical training. Mil Med.

[6] Suner S, Simmons W, Savitt DL (2000). A porcine model for instruction of lateral canthotomy. Acad Emerg Med.

[7] Phillips L, Stack L, Thurman RJ (2015). Addressing ophthalmology education for newly matriculated emergency medicine residents using innovative models. Simul Healthc.

